# Role of plasmacytoid dendritic cells in humoural and CD8^+^ T-cell memory responses following BNT162b2 mRNA vaccination: a preclinical mouse study

**DOI:** 10.1016/j.ebiom.2026.106432

**Published:** 2026-08-21

**Authors:** Chiara Pizzichetti, Irene Latino, Tommaso Virgilio, Arianna Capucetti, Kamil Chahine, Luciano Cascione, Simone G. Moro, Giorgia Bilato, Melanie Brügger, Nedim Kozarac, Alain Pulfer, Louis Renner, Roshan S. Thakur, Charaf Benarafa, Daniel F. Legler, Santiago F. González

**Affiliations:** a Faculty of Biomedical Sciences, Institute for Research in Biomedicine, Università Della Svizzera Italiana, Bellinzona, Switzerland; b Graduate School for Cellular and Biomedical Sciences, University of Bern, Bern, Switzerland; c Faculty of Biomedical Sciences, Institute of Oncology Research, Università Della Svizzera Italiana, Bellinzona, Switzerland; d Institute of Virology and Immunology, Bern and Mittelhäusern, Bern, Switzerland; e Department of Infectious Diseases and Pathobiology, Vetsuisse Faculty, University of Bern, Bern, Switzerland; f Multidisciplinary Center for Infectious Diseases, University of Bern, Bern, Switzerland; g Institute of Cell Biology and Immunology Thurgau (BITG) at the University of Konstanz, Kreuzlingen, Switzerland; h Theodor Kocher Institute, University of Bern, Bern, Switzerland; i Department of Biology, University of Konstanz, Konstanz, Germany; j Immunology and General Pathology Laboratory, Department of Biotechnology and Life Sciences, University of Insubria, Varese, Italy; k Unit of Molecular Pathology, Biochemistry and Immunology, Istituto di Ricovero e Cura a Carattere Scientifico (IRCCS) MultiMedica, Milan, Italy

**Keywords:** Plasmacytoid dendritic cells, CD8+ T cells, SARS-CoV2-immunity, COVID-19 vaccines, mRNA vaccine, BNT162b2

## Abstract

**Background:**

The Pfizer-BioNTech coronavirus vaccine (BNT162b2) was among the first nanoparticle-based vaccines approved by the World Health Organisation (WHO) and demonstrated 95% efficacy against Severe Acute Respiratory Syndrome Coronavirus 2 (SARS-CoV-2) infection. Despite its success, the precise immune mechanism behind its effectiveness remains poorly understood. This study investigated the early immune responses occurring in the draining lymph node (dLN) following vaccination.

**Methods:**

A well-established murine vaccination model was used to investigate the distribution and immunological effects of BNT162b2 *in vivo*. Vaccine trafficking to the dLN, cellular uptake, and spike protein expression were assessed following immunisation. Single-cell transcriptomic analyses and functional *in vivo* experiments were performed to identify the immune cell populations involved in orchestrating vaccine-induced responses and to characterise their interactions with adaptive immune cells.

**Findings:**

BNT162b2 was rapidly transported to the dLN, where it was predominantly captured by leukocytes that subsequently expressed the SARS-CoV-2 spike protein. Among these cells, plasmacytoid dendritic cells (pDCs) emerged as regulators involved in both inflammatory and humoural immune responses. Single-cell transcriptomic profiling revealed active interactions between pDCs and CD8^+^ T cells. Functional *in vivo* studies further demonstrated that pDCs promoted CD8^+^ T-cell activation and expansion, indicating an involvement in shaping adaptive immunity following vaccination.

**Interpretation:**

These findings identify pDCs as antigen-presenting cells involved in coordinating the immune response to BNT162b2. By regulating both innate and adaptive immune pathways and facilitating CD8^+^ T-cell activation, pDCs appear to play a functional role in the efficacy of this mRNA vaccine. This study provides additional insights into the immunological processes underlying mRNA vaccine-induced protection and may inform the future design and optimisation of mRNA-based immunotherapies and vaccines.

**Funding:**

Swiss National Science Foundation, Leonardo Foundation.

**Research in context**

**Evidence before this study**

We conducted bibliographic searches throughout the course of this study using PubMed. Searches were performed from database inception until May, 2026, using combinations of the terms: “BNT162b2” OR “mRNA vaccine” OR “COVID-19 vaccine”; “draining lymph node” OR “lymph node”; “plasmacytoid dendritic cell” OR “pDC” OR “dendritic cell”; “immune response” OR “antigen presentation”. We also screened the reference lists of relevant articles. Previous studies have established the high efficacy of BNT162b2 and demonstrated that mRNA vaccines induce robust innate and adaptive immune responses. Studies have shown that lipid nanoparticle-formulated mRNA vaccines reach draining lymph nodes and are taken up by immune cells, promoting antigen expression and activation of vaccine-specific T-cell and antibody responses. Plasmacytoid dendritic cells have been implicated in antiviral immunity and type I interferon production; however, their contribution to the early cellular events occurring in the draining lymph node following BNT162b2 vaccination has remained incompletely characterised *in vivo*.

**Added value of this study**

Using a murine model of vaccination, we characterised the early immune response to BNT162b2 within draining lymph nodes. We show that vaccine-associated material rapidly reaches the draining lymph node and is predominantly captured by leukocytes expressing the SARS-CoV-2 spike protein. Through single-cell transcriptomics and *in vivo* functional approaches, we identified plasmacytoid dendritic cells as contributors to the inflammatory and adaptive immune responses elicited by vaccination. Our data further indicate interactions between plasmacytoid dendritic cells and CD8^+^ T cells during the early stages of the immune response and are consistent with a role for these cells in promoting CD8^+^ T-cell activation and expansion.

**Implications of all the available evidence**

Together with previous studies demonstrating the importance of innate immune activation for mRNA vaccine efficacy, our findings provide additional evidence regarding the cellular mechanisms operating in draining lymph nodes after BNT162b2 vaccination. These results support the involvement of plasmacytoid dendritic cells in shaping vaccine-induced immune responses in mice and may help guide future studies aimed at optimising mRNA vaccine platforms and adjuvant strategies. Further investigation in human systems will be required to determine the translational relevance of these observations.

## Introduction

The emergence of symptomatic coronavirus disease 2019 (COVID-19) across the planet triggered a global race for the development of an effective vaccine. Building on existing discovery and preclinical data from SARS-CoV and MERS-CoV vaccine studies, different candidates were developed including recombinant protein-based vaccines, inactivated and live-attenuated SARS-CoV-2-based vaccines, various vector-based platforms, and innovative nucleic acid-based approaches.^1^^,^^2^ Among these, RNA vaccines showed remarkable results in preclinical studies and accelerated clinical trials, and two of them, the Comirnaty-BNT162b2 (Pfizer-BioNTech) and the Spikevax (Moderna), were approved and have been used extensively.^1^^,^^3^, ^4^, ^5^ BNT162b2 was the first RNA vaccine authorised, demonstrating 95% efficacy in preventing COVID-19.^4^^,^^6^ Since then, Pfizer-BioNTech has released updated RNA vaccines to address the need for protection against new circulating SARS-CoV-2 variants.^6^ However, despite its success, the immunological mechanisms driving the efficacy of these newly approved therapies remain incompletely elucidated.^7^ While these vaccines are designed to deliver nucleoside-modified mRNA encoding viral spike protein, the *in vivo* cellular and molecular events that translate mRNA delivery into robust and durable adaptive immunity are not fully defined.

The principal route of administration for most vaccines, including the Pfizer-BioNTech coronavirus vaccine, is intramuscular (i.m.) injection. Following administration, the vaccine can reach the draining lymph node via lymphatic drainage or intracellular transport associated with antigen-presenting cells.^8^ The specific pathway of lymph node access is known to shape the immune response, influencing both the magnitude and quality of immune cell activation and targeting.^9^^,^^10^ While clinical data have demonstrated that Pfizer-BioNTech administration induces local inflammation, lymphocyte infiltration, and spike protein expression at the injection site,^11^, ^12^, ^13^ the precise mechanism driving vaccine trafficking to the lymph node remains unclear. Moreover, recent studies have analysed the immune response to the BNT162b2 vaccine in humans and mice. Kariko and colleagues discovered that the m1Ψ-modification on RNA reduces the sensing of the mRNA by most Toll-like receptors.^14^^,^^15^ However, the vaccine successfully activated the melanoma differentiation-associated protein 5 (MDA5) in macrophages and dendritic cells. This resulted in triggering the interferon type I receptor (IFNAR1) signalling pathway, which is needed to activate the adaptive immune system.^16^

This study aims to dissect the early immunological events following *in vivo* Pfizer-BioNTech coronavirus vaccination, focusing on the role of plasmacytoid dendritic cells (pDC) in orchestrating both humoral and cell-mediated immune responses.

## Methods

### Ethics statement

All animal procedures were conducted in accordance with the Swiss Federal Veterinary Service, and the protocols (animal permits TI 33/2021, TI 87/2024) were approved by the Animal Welfare Committee (Commissione Cantonale per gli Esperimenti sugli Animali) of the Cantonal Veterinary Office.

### Mice

C57BL/6J mice were bred in-house or purchased from Charles River Laboratories. B6.129P2(Cg)-Cx3cr1tm1Litt/J (CX3CR1-GFP) mice were initially purchased from Jackson Laboratories and subsequently bred in our animal facility at the Institute of Research in Biomedicine (IRB). B6.129(ICR)-Tg (CAG-ECFP)CK6Nagy/J (CK6-ECFP) mice were initially purchased from Charles River Laboratories and subsequently bred in our animal facility at the IRB. The following transgenic strains were used: CD169^DTR^,^17^ CD11c^DTR/GFP^,^18^ IFNAR^−/−^.^19^ Mice used for experiments included equal numbers of males and females, aged 6–12 weeks at the time of vaccination, in good health, and with no abnormal clinical signs. Mice were bred under specific pathogen-free (SPF) conditions in individually ventilated cages, with a controlled light-dark cycle (12:12), room temperature (20–24 °C), and relative humidity (30%–70%). All the experiments were run in the basics of Biosafety Level 2 (BSL2) facility.

### Vaccination and injections

Discarded remnant material (Pfizer-BioNTech, BNT162b2) was stored at −80 °C at the final concentration of 100 μg/ml. For fluorescence-based techniques, the vaccine was labelled with DiD (#V22889, Invitrogen) as previously described.^20^ The final concentration was checked at the nanodrop, and the vaccine was diluted back to the initial concentration.

Pfizer-BioNTech mRNA vaccine (2 μg in 10 μl per mouse) was injected s.c. into each footpad or i.m. into each calf of isoflurane-anesthetised mice (2% volume-to-volume, Baxter), and the popliteal lymph node (pLN) was collected at different time points. For macrophage depletion, CD169^DTR^ mice were depleted 2 days before and 4 days after vaccination with 200 ng of diphtheria toxin (Sigma–Aldrich) intraperitoneally (i.p.) and subcutaneously (s.c.) in the footpad. For conventional dendritic cell (cDC) depletion, CD11c^DTR/GFP^ mice were depleted 2 days before and 4 days after vaccination by injecting 80 ng of diphtheria toxin (Sigma–Aldrich) s.c. in the footpad. For pDCs depletion, 400 μg/mouse i.p. (day −1, +3, +7) and 100 μg/footpad s.c. (day −1, +1, +3, +5, +7, +9) of αCD317 (clone 927, BioXCell) was administered to the mice. The depletion protocol was established based on previously published protocols,^21^^,^^22^ and the timing of the challenge injection was optimised based on pDCs recovery kinetics (Supplementary Fig. 2A).^21^^,^^22^

### Two-photon intravital microscopy

The day before the surgery, mice were injected with 500 ng αCD21/35 Pacific Blue s.c. in the footpad. On the day of the surgery, mice were injected with 500 ng of αCD169 APC s.c. in the footpad. Mice were anesthetised with an i.p. injection of a cocktail containing ketamine (100 mg/kg, Sintetica) and xylazine (10 mg/kg, Virbac). The mouse was positioned on a microscopic stage. The right foot was maintained and extended by fastening the fingers using a suture thread, and fasteners were attached to the hip. Finally, the pLN was exposed through an incision in the skin and by removing the overlying fat tissue. Mice were then injected s.c. in the footpad with 2 μg of DiD-labelled BNT162b2 vaccine. The imaging of the pLN was performed on a customised up-right two-photon platform (TrimScope, LaVision BioTec) as previously described.^23^ The objective used was a 10×, and two-photon micrographs were acquired every 30 s for a total duration of 90 min. Analyses were performed using the Imaris 9.9.1 software (Oxford Instruments) and ImageJ.^24^

### Expression and purification of YFP-tagged RBD and RBD

Expression and purification of YFP-tagged RBD and RBD were performed with modifications to the methods described by Bierig et al.^25^ A DNA sequence encoding YFP-RBD-His was synthesised and cloned into the pcDNA3.1 (+) vector by GenScript. Expi293F cells (#A14527, ThermoFisher) were maintained according to Good Laboratory Practice (GLP) procedures to prevent cross-contamination and were regularly tested for mycoplasma contamination and confirmed to be mycoplasma-free. Formal cell-line authentication was not performed. The cells were cultured in Expi293 Expression Medium (#A1435101, ThermoFisher) in an orbital shaker at 120 rpm, 37 °C, and 8% CO_2_. On the day of transfection, cells were adjusted to 3.0 × 10^6^ cells/mL and transiently transfected with 1 μg/mL pcDNA3.1 (+) YFP-RBD-His plasmid DNA and 2.7 μg/μl DNA ExpiFectamine™ 293 Reagent (#A14524, ThermoFisher). Twenty-two hours post-transfection, ExpiFectamine™ enhancers 1 (#A14525, ThermoFisher) and 2 (#A14526, ThermoFisher) were added according to the manufacturer’s instructions. One week after transfection, the culture supernatant was supplemented with 10 mM imidazole (#I2399, Sigma–Aldrich), 300 mM NaCl (#S7653, Sigma–Aldrich), and 50 mM Tris (pH 7.5) (#T1503, Sigma–Aldrich), and centrifuged at 2000 *g* for 30 min at 4 °C. The clarified supernatant was loaded onto a HisTrap excel HP column (#17-3712-05, Cytiva) pre-equilibrated with binding buffer (50 mM Tris, pH 7.5, 10 mM imidazole, and 300 mM NaCl) using an AKTA purifier 10 FPLC system (Cytiva). The column was washed with five bed volumes of wash buffer (50 mM Tris, pH 7.5, 30 mM imidazole, and 300 mM NaCl), and proteins were eluted with three bed volumes of elution buffer (25 mM HEPES, pH 7.4, 300 mM NaCl, and 300 mM imidazole). Eluted peak fractions were pooled and dialysed overnight in PBS (#D8537, Sigma–Aldrich). The dialysed protein was concentrated to 2 mL using Amicon® Ultra Centrifugal filters (10 kDa cutoff, #UFC801024, Millipore). The concentrated protein was then injected into a Superdex 200 16/600 column (#28-9893-35, Cytiva) pre-equilibrated with PBS. Peak fractions were collected, concentrated using Amicon® Ultra Centrifugal filters, filter-sterilised with 0.2 micron Millex® syringe filters (#SLGV033RS, Millipore Sigma), and stored in small aliquots at −80 °C. For the production of RBD protein without the YFP tag, all steps were identical except that after Ni-NTA purification, the dialysed sample was treated with PreScission Protease (#27-0843-01, Cytiva) at a 1:100 ratio for 6 h at 4 °C, and then passed through a GST resin (#17-5132-01, Cytiva) to remove the PreScission Protease. The flow-through was re-passed through the Ni-NTA resin to remove the cleaved YFP tag. As described above, the Ni-NTA eluted fractions were collected, concentrated, and injected into the Superdex 200 16/600 column for final purification.

### Serum neutralisation test

Mouse sera were incubated at 56 °C for 30 min for complement inactivation. SARS-CoV-2^(S−D614G)^ (100 TCID_50_) inoculum was incubated with two-fold serial dilutions of mouse serum (starting at 1:20) for 60 min at 37 °C. The mixtures were then added to confluent Vero/TMPRSS2 cell monolayers in 96-well culture plates in quadruplicate wells and incubated for 72 h at 37 °C as described previously.^26^ Vero/TMPRSS2 cells were maintained according to Good Laboratory Practice (GLP) procedures to prevent cross-contamination and were regularly tested for mycoplasma contamination and confirmed to be mycoplasma-free. Formal cell-line authentication was not performed. The neutralising dose 50% (ND_50_) values were calculated according to the Spearman–Kärber method^27^ and data were analysed by Mann Whitney U test.

### Tissue collection and preparation of single-cell suspension

Mice were sacrificed with CO_2,_ and organs were harvested. For LNs, after collection, they were disrupted with tweezers and filtered with a 40 μm cell strainer. For spleens, after collection, they were disrupted on a 40 μm cell strainer, and red blood cells (RBCs) were lysed using the Lysing Buffer (#555899, BD Biosciences). Then, CD8^+^ T cells or pDCs were isolated using the EasySep™ mouse CD8^+^ T cell isolation kit (#19853, StemCell) and the mouse Plasmacytoid dendritic cell isolation kit (#130-107-093, Miltenyi). For lungs, mice were perfused with PBS, and lungs were processed as previously described.^28^ RBCs were lysed, and lymphocytes were isolated from the lungs using a 40/80 gradient Percoll (VWR International), followed by centrifugation at 1250 rcf for 20 min without acceleration and break.

### Flow cytometry analysis of vaccine capture and spike expression

pLNs were processed as previously described. Single-cell suspensions were stained with antibodies listed in Supplementary Table S1. Fc receptors were blocked with αCD16/32, and dead cell exclusion was performed using the Zombie Aqua™ Fixable Viability Kit (Biolegend, San Diego, USA). Surface staining was carried out for 30 min, and vaccine capture was detected as DiD^+^ cells, while spike expression was detected using αSpike antibody. All operations were done at 4 °C in the dark. B cells were identified as CD45^+^, CD3^-^, B220^+^, PDCA^−^. pDCs were identified as CD45^+^, CD3^-^, B220^+^, PDCA^+^. T cells were identified as CD45^+^, B220^-^, CD3^+^. In the CD45^+^, CD3^-^, B220^-^, all the downstream cells were gated. Neutrophils/monocytes were identified as GR1^+^. Medullary macrophages were identified as GR1^-^, F4/80^+^, and CD11b^+^. NK cells were identified as GR1^-^, F4/80^-^, and NK1.1^+^. DCs were identified as GR1^-^, F4/80^-^, NK1.1^-,^ and two major subpopulations were distinguished based on CD11b expression: CD11b^+^ cDCs and CD11b^−^ cDCs. Subcapsular sinus macrophages were identified as GR1^-^, F4/80^-^, NK1.1^-^, CD11c^−^, CD11b^+^. All samples were acquired on fluorescence-activated cell sorting (FACS) Symphony A3 flow cytometer (BD Biosciences, New Jersey, USA) and were analysed with FlowJo software version 10.7.1 (FlowJo LLC, Ashland, Oregon).

### Flow cytometry analysis of memory B cells

SARS-CoV-2 RBD-YFP protein was produced in-house, as described above.^25^ pLNs were processed as previously described. Single-cell suspensions were stained with antibodies listed in Supplementary Table S1. Fc receptors were blocked with αCD16/32, and dead cell exclusion was performed using the Zombie Aqua™ Fixable Viability Kit (Biolegend, San Diego, USA). Surface staining was carried out for 30 min. All operations were done at 4 °C in the dark. RBD^+^ memory B cells were identified as CD45^+^, CD3^-^, B220^+^, CD43^-^, CD19^+^, CD1D^−^, IgG^+^, RBD^+^. All samples were acquired on fluorescence-activated cell sorting (FACS) Symphony A3 flow cytometer (BD Biosciences, New Jersey, USA) and were analysed with FlowJo software version 10.7.1 (FlowJo LLC, Ashland, Oregon).

### Flow cytometry data analysis with crusty

pLNs were processed as previously described. Samples were transferred in a 96-well plate, and they were stimulated with a mix of 50 μg/ml brefeldin (#B7651, Sigma–Aldrich), 20 ng/ml phorbol myristate acetate (PMA) (#10933, Sigma–Aldrich) for 3 h at 37 °C. Single-cell suspensions were stained with antibodies listed in Supplementary Table S1. Fc receptors were blocked with αCD16/32, and dead cell exclusion was performed using the Zombie Aqua™ Fixable Viability Kit (Biolegend, San Diego, USA). Surface staining was carried out for 30 min at 4 °C. Samples were then fixed and permeabilised in eBioscience™ Foxp3/Transcription Factor Staining Buffer Set (#00-5523-00, ThermoFisher) and stained intranuclearly with αKi-67 PE in Fix/Perm buffer 30 min at 4 °C. T cell subsets were distinguished based on the expression of CD4 and CD8 markers. For cluster discovery, the gates were downsampled to have 2900 CD8^+^ T cells per sample. Files were then exported as.csv files and imported to CRUSTY web platform. Clusters were determined by applying the PhenoGraph algorithm.^29^ To study the identified clusters, CD8^+^ T cell subsets were gated as CD45^+^, B220^-^, CD3^+^, CD8^+^, in which the following subsets were identified: Proliferating Naïve CD8^+^ T cells Ki-67^+^, CD62L^+^, CD44^-^; Proliferating CM CD8^+^ T cells Ki-67^+^, CD62L^+^, CD44^+^; Naïve CD8^+^ T cells Ki-67^-^, CD69^-^, CD62L^+^, CD44^−/+^; DN CD8^+^ T cells CD8^+^, Ki-67^-^, CD69^-^, CD62L^−^, CD44^−/+^; Active Naïve CD8^+^ T cells Ki-67^-^, CD69^+^, CD62L^+^, CD44^-^; Active CM CD8^+^ T cells Ki-67^-^, CD69^+^, CD62L^+^, CD44^+^; Active DN CD8^+^ T cells Ki-67^-^, CD69^.^, CD62L^−^, CD44^−/+^. All samples were acquired on fluorescence-activated cell sorting (FACS) Symphony A3 flow cytometer (BD Biosciences, New Jersey, USA) and were analysed with FlowJo software version 10.7.1 (FlowJo LLC, Ashland, Oregon).

### Flow cytometry analysis of tissue-resident memory T cells in the lungs

Lungs were processed as described above. Then, lymphocytes were isolated with percoll gradient. Fc receptors were blocked with αCD16/32, and dead cell exclusion was performed using the Zombie Aqua™ Fixable Viability Kit. Surface staining was carried out for 30 min at 4 °C. T cells were identified as CD45^+^, B220^-^, CD3^+^. Then CD4^+^ and CD8^+^ T cells were identified, and tissue-resident cells were gated as CD69^+^. To conclude, SARS-CoV-2 specific memory T cells were identified as CD45^+^, B220^-^, CD3^+^, CD8^+^, CD69^+^, Class I tetramer^+^. All samples were acquired on fluorescence-activated cell sorting (FACS) Symphony A3 flow cytometer (BD Biosciences, New Jersey, USA) and were analysed with FlowJo software version 10.7.1 (FlowJo LLC, Ashland, Oregon).

### Multiplex assay

pLNs were collected at different time points after vaccination and carefully disrupted in 75 μL of cold PBS^−/−^to avoid cell rupture. The suspension was centrifuged at 1500 rpm for 5 min, and the supernatant was collected and stored at −80 °C until the usage. According to the manufacturer’s instructions, the concentration of cytokines in the lymph was determined by LEGENDPlex assays (#740150, Mouse Inflammation Panel (13-plex); #740622, Mouse Anti-Virus Response Panel (13-plex); Biolegend). Samples were analysed using a Symphony A3 flow cytometer (BD Biosciences, New Jersey, USA), and data were analysed using LEGENDPlex software (BioLegend).

### ELISA

SARS-CoV-2 RBD protein was produced in-house, as described above. Blood was collected from the tail vein in capillary blood collection system tubes (GK 150 SE Gel 200 μl, Kabe Labortechnik) on day 7 and day 10 p.v. Blood was then centrifuged at 3,000 *g* for 10 min RT. The serum was aliquot into 1.5 ml Eppendorf tubes and stored at −80 °C until the usage. The experiment was then performed as previously described. Half-well Nunc ELISA plates were coated with 5 μg/ml SARS-CoV-2 RBD protein in PBS^−/−^ buffer and incubated overnight at 4 °C. The experiment was then performed as previously described.^20^

### ELISpot

For enzyme-linked immunosorbent spot assay (ELISpot), on day 10 p.i., popliteal LNs were removed aseptically, disrupted, and passed through a 40-μm cell strainer. 4 × 10^5^ cells were plated on SARS-CoV-2 RBD-coated (5 μg/ml) filter plates (MultiScreen HTS, Merck Millipore) and incubated overnight at 37 °C. For detection, a biotin-conjugated anti-IgG or anti-IgM was used, followed by streptavidin-conjugated horseradish peroxidase (HRP). A developing solution consisting of 200 μL 3-amino-9-ethylcarbazole (AEC) solution (Sigma–Aldrich) in 9 mL sodium acetate buffer containing 4 μL 30% H_2_O_2_ was subsequently added. Spots were read on a CTL ELISPOT reader using ImmunoSpot 5.1 software (Cellular Technology).

### *In vitro* pDCs pulsing with DiD-labelled BNT162b2 vaccine

1 μg/ml DiD-labelled BNT162b2 vaccine was incubated in complete RPMI 1640 (#31870-025, Gibco) supplemented with 10% heat-inactivated foetal bovine serum (FBS) (#10270-106, Gibco), 50 units/ml penicillin +50 μg/ml streptomycin (#15070-063, Gibco), 1% glutamax (#35050-038, Gibco), 1% sodium pyruvate (Gibco), 1% nonessential amino acids (#11140-035, Gibco) for 60 min at 37 °C with 5% CO^2^ to allow the formation of the protein corona. pDCs were isolated as previously described. 3 × 10^5^ cells/ml pulsed pDCs or naïve pDCs were plated in a Petri dish with 1 μg/ml DiD-labelled BNT162b2 vaccine or complete RPMI alone for 3 h at 37 °C.

### Immunofluorescence

For mouse microscopy experiments, pLNs were harvested at different time points and fixed in 4% paraformaldehyde (PFA; Merck-Millipore) for 12 h at 4° and then embedded in 4% Low Gelling Temperature Agarose (Sigma–Aldrich). Sequential sections of 40 μm were cut with a vibratome (VT1200S, Leica Microsystems) and stained in a blocking buffer composed of PBS supplemented with calcium and magnesium (PBS+/+), TritonX100 (VWR) 0.1%, BSA 5% (VWR), and fluorescently labelled antibodies at the appropriate concentration. After overnight incubation at RT, samples were washed in PBS+/+ with 0.05% Tween 20 (Sigma–Aldrich), followed by washes with PBS−/−, and mounted on glass slides. To study the vaccine uptake and Spike expression, CX3CR1-GFP mice were immunised as described in the “Vaccination and injections” section, and slices were stained for CD21/35 and spike. pLN regions were manually identified based on CX3CR1 and CD21/35 expression.

To study pDC-T cell interaction, pDCs and CD8^+^ T cells were isolated as reported in the “Tissue collection and preparation of single-cell suspension” section from WT and CK6-ECFP mice, respectively. pDCs were then stimulated and pulsed as previously described, and naïve and pulsed pDCs were labelled with 1 μM CellTrace™ CFSE Cell Proliferation kit (#C34554, ThermoFisher) and 1 μM CellTracker™ Deep Red (#C34565, ThermoFisher) respectively. 3 × 10^6^ naïve and pulsed pDCs and 4 × 10^6^ CD8^+^ T cells were injected intravenously in the mice. pLN slices were stained for CD169. Immunofluorescence confocal images were acquired using a Leica TCS SP5 confocal microscope (Leica Microsystems), and images were analysed using ImageJ^24^ and Imaris 9.7.2 Cell Imaging Software (Oxford Instruments). The antibodies used are listed in Supplementary Table S1.

### scRNA-seq data analysis

Public data from the study, which included one draining iliac lymph node per experimental condition, were retrieved from GEO (accession number GSE179131) and analysed using Seurat (v5.1.0).^30^ Cells with fewer than 200 or more than 5000 genes or mitochondrial RNA content exceeding 8% were excluded from the analysis. Genes expressed in very few cells (less than 3) were discarded. Raw count data were log normalised with a scaling factor of 10,000. Subsequently, the most variable features were identified using the variance stabilising transformation method, selecting the top 2000 features based on their variability across the dataset. Finally, the data were scaled to mean centre and unit variance for each feature, utilising all genes in the dataset. We utilised Seurat’s anchor-based integration method on the dataset. Cell types were annotated with the ImmGen database.^31^ TFH cells were manually annotated using marker genes retrieved from PanglaoDB.^32^ Markers were selected based on ubiquitousness index <0.01 and by excluding genes with zero specificity in mouse. A TFH genes signature score was computed using Seurat function AddModuleScore, and the cluster exhibiting the highest score was identified and therefore annotated as TFH.

### Cell communication analysis

To infer intercellular communication between cell populations of interest, we employed CellChat (v1.5.0),^33^ a computational framework that predicts ligand-receptor interactions from scRNA-Seq data. The normalised gene expression matrix from Seurat was imported into CellChat using the createCellChat function. The CellChatDB.mouse database was used to identify potential ligand-receptor pairs specific to the mouse model. The communication probability was computed using a probabilistic model, and key signalling pathways were inferred using computeCommunProb and computeCommunProbPathway functions. The strength and directionality of interactions between cell populations were visualised using netVisual_circle, netVisual_bubble, and netAnalysis_contribution functions. The top ligand-receptor interactions were further examined in the context of known cellular functions and disease-related pathways. Statistical significance for differential ligand-receptor interactions was assessed using permutation-based significance testing implemented in CellChat. Comparisons between groups were performed using the Wilcoxon rank-sum test, and p-values were adjusted for multiple comparisons using the Benjamini-Hochberg method. A significance threshold of p < 0.05 was applied.

### Statistical analysis

All statistical analyses were performed with Prism (GraphPad Software v10.4.1). For two-group comparisons, p values were determined using Student’s t-tests (two-tailed). For more than two groups, a comparison of one-way or two-way ANOVAs followed by Bonferroni correction was applied. Differences between groups were considered significant for p values < 0.05. Sample sizes were predetermined based on prospective power calculations assuming a two-sided α level of 0.05 and effect sizes derived from previous experiments using the same vaccination model. These analyses indicated that five animals per group were sufficient to detect biologically meaningful differences with adequate statistical power while minimising animal use. Animals were randomly assigned to experimental groups. Only healthy mice (6–12 weeks old and free of abnormal clinical signs) were included in the study. Investigators were not blinded to group allocation.

### Role of funders

The funders had no role in study design, data collection, data analysis, data interpretation, writing of the manuscript, or the decision to submit the work for publication.

## Results

### Pfizer-BioNTech coronavirus vaccine is rapidly transported to the draining lymph node, accumulates in the medullary region, and initiates an IFN-mediated inflammatory response

**Fig. 1:**
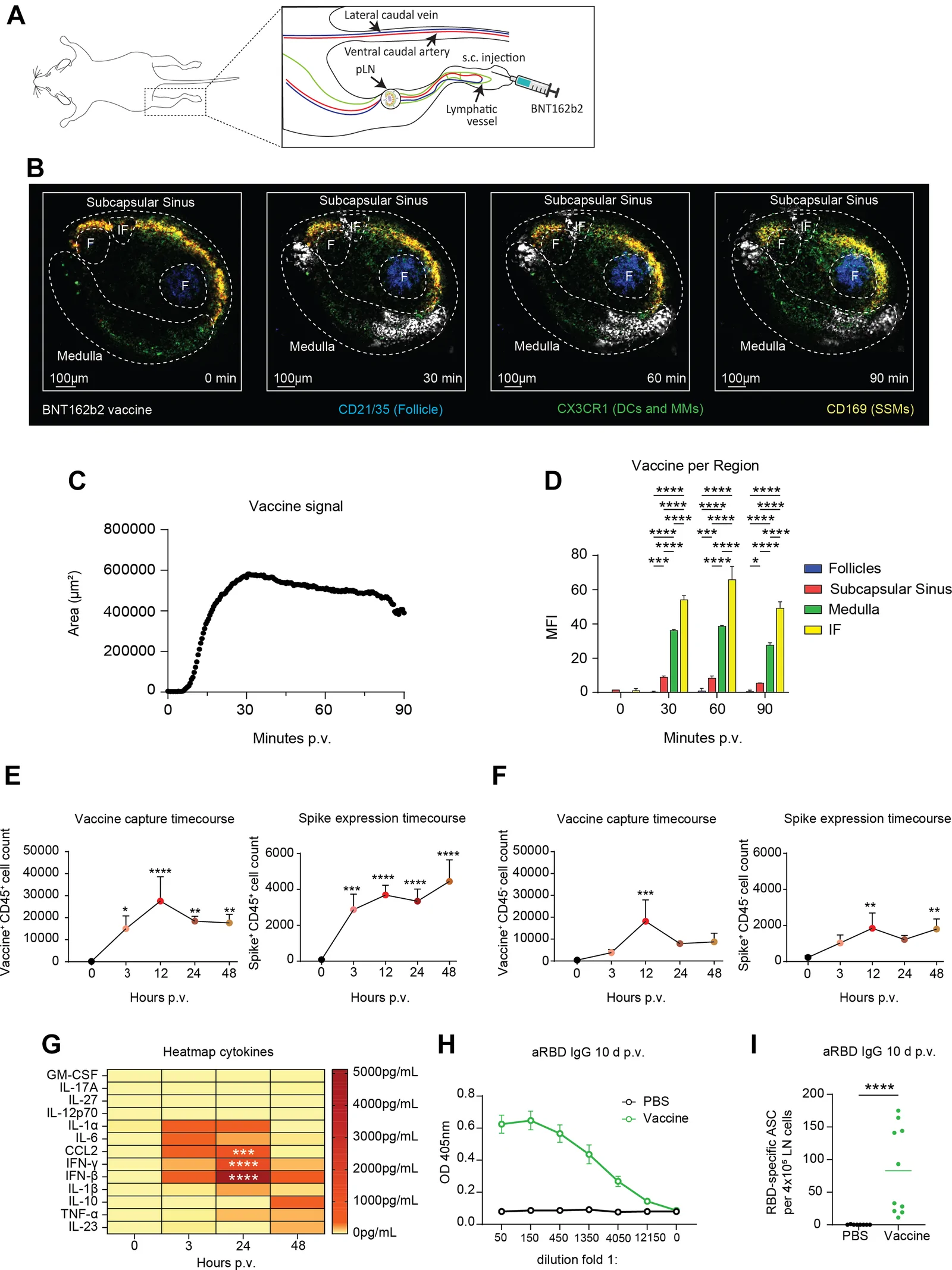
**BNT162b2 vaccine has a rapid arrival in the dLN, where it gets captured by immune cells. (A)** Experimental model of subcutaneous (s.c.) injection of the BNT162b2 vaccine in the footpad. **(B)** Representative intravital 2-photon micrographs acquired at 0, 30, 60, and 90 min p.v. The micrographs show serial intravital images of the same LN acquired over time. IF stands for Interfollicular region; DCs, Dendritic cells; MMs, Medullary Macrophages; SSM, Subcapsular Sinus Macrophages. **(C)** Time course showing the arrival of the BNT162b2 vaccine in the pLN during the first 90 min p.v. **(D)** Quantification of the mean fluorescence intensity (MFI) of the vaccine in the different areas of the LN at 0, 30, 60, and 90 min p.v. **(E)** Capture of labelled BNT162b2 vaccine (left) and expression of coronavirus spike protein (right) by immune cells in the pLN at indicated time points p.v. Statistical analyses compare each time point to the 0 h p.v. **(F)** Capture of labelled BNT162b2 vaccine (left) and expression of coronavirus spike protein (right) by not immune cells in the pLN at indicated time points p.v. Statistical analyses compare each time point to the 0 h p.v. Differences not reaching statistical significance are represented by the absence of asterisks. **(G)** Heatmap showing the lymph expression of different cytokines in the pLN at different time points p.v. Differences not reaching statistical significance are represented by the absence of asterisks. **(H)** Anti-SARS-CoV-2 RBD IgG titres measured by ELISA at day 10 p.v. Statistical analyses compare each time point to the 0 h p.v. **(I)** Local anti-SARS-CoV-2 RBD IgG response measured by ELISPOT at day 10 p.v. **(E, F)** n = 3 for 0 group, n = 6 for all the other groups. Data are presented as mean ± SD. Two-way ANOVA **(D, G)** and One-way ANOVA **(E, F)** were followed by Bonferroni correction for multiple comparisons **(I)** Mann–Whitney U test. (∗p < 0.05, ∗∗p < 0.01, ∗∗∗p < 0.001 ∗∗∗∗p < 0.0001).

To assess both the local and the systemic immune responses against the BNT162b2 vaccine, we immunised C57BL/6 mice via subcutaneous (s.c.) injection in the footpad (Fig. 1A, Supplementary Fig. 1A). We administered a dosage of 2 μg/mouse, which has been shown to elicit immune responses comparable to those observed in humans.^16^ The transport dynamics of the vaccine in real-time from the injection site to the draining popliteal lymph node (pLN) were analysed by two-photon intravital microscopy. We observed a rapid arrival (of the labelled vaccine 10 min post-vaccination (p.v.)), which peaked at 30 min p.v. (Fig. 1C), and accumulated primarily in the interfollicular (IF) and medullary regions of the pLN (Fig. 1B, D; Supplementary Movie 1). Subsequently, we conducted a time course study, monitoring the vaccine capture and the expression of the spike protein in leukocytic and non-leukocytic cells by flow cytometry. Remarkably, vaccine uptake and spike protein expression were already detectable 3 h p.v., with vaccine capture peaking at 12 h p.v. while the percentage of spike-expressing cells continued to increase up to 48 h p.v. respectively (Fig. 1E and F; Supplementary Fig. 1B and C). The analysis of CD45^-^ and CD45^+^ cells showed similar dynamics, with leukocytes being the primary contributors to vaccine uptake and spike protein expression (Fig. 1E and F). Next, we investigated the immune response by monitoring inflammatory and antibody production following vaccination. In line with previous studies,^16^ our model induced a strong inflammatory response, correlating with an increase in the early expression of interferon signalling at 24 h p.v. (Fig. 1G). Furthermore, the vaccine triggered a robust local and systemic humoural response, marked by a significant increase in the anti-SARS-CoV-2 spike receptor binding domain (RBD) IgM titres at 7 and 10 days p.v. (Supplementary Fig. 1D and E) and the IgG levels at 10 days p.v. (Fig. 1H and I). compared to non-vaccinated controls.

### Plasmacytoid dendritic dells contribute to the humoural response to BNT162b2 vaccination

**Fig. 2:**
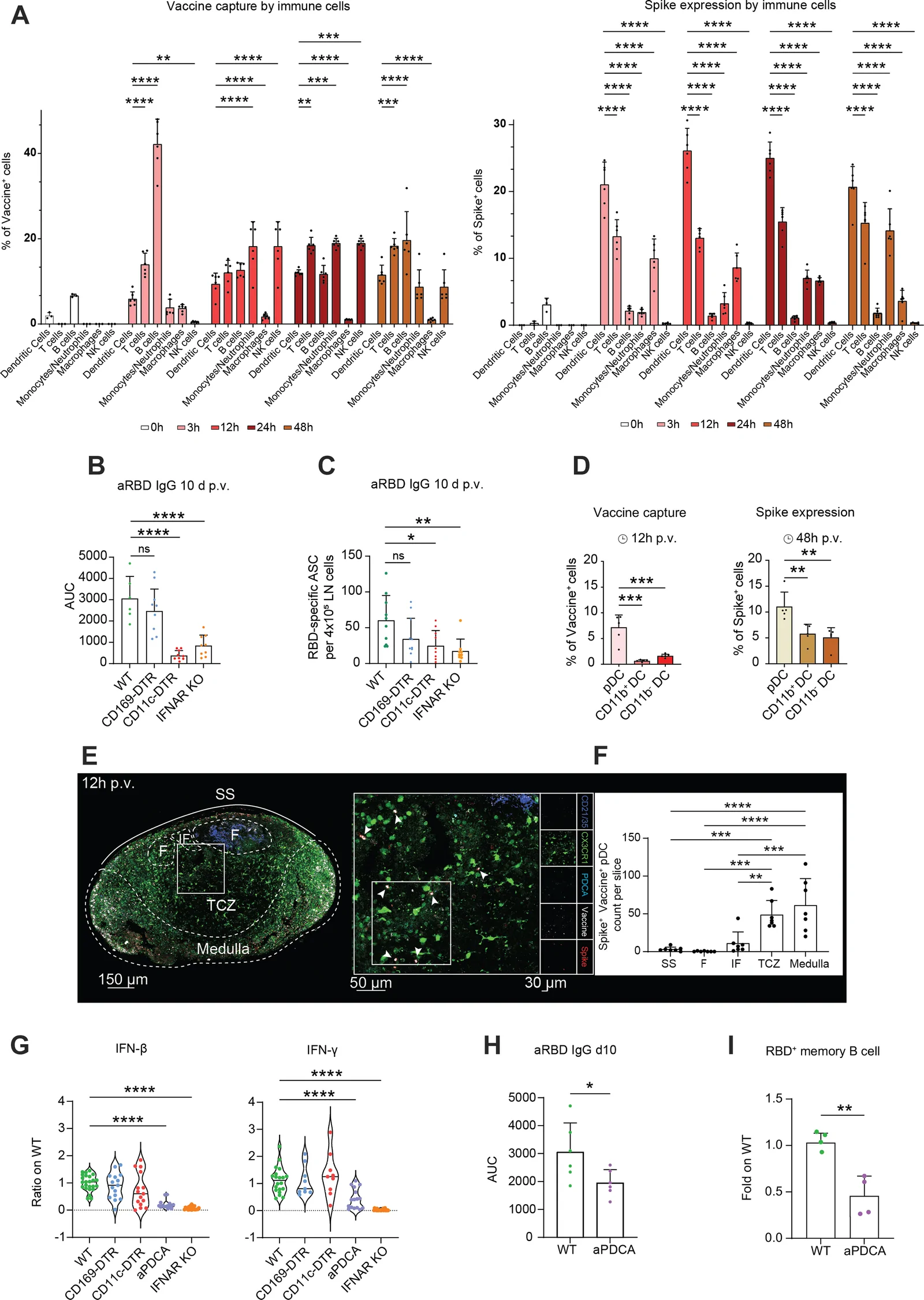
**pDCs contribute to the humoural response following BNT162b2 vaccination. (A)** Histogram showing the frequency of immune cells positive for the BNT162b2 vaccine (left) and the coronavirus spike protein (right) in the pLN at different time points p.v. (n = 3 for 0 h group, n = 6 for all the other groups). **(B)** Area under the curve (AUC) indicating anti-SARS-CoV-2 RBD IgG titre for WT, CD169-DTR, CD11c-DTR, and IFNAR KO mice at day 10 p.v. (n = 6 for WT group, n = 9 for all the other groups. **(C)** Local anti-SARS-CoV-2 RBD IgG response measured by ELISPOT in WT, CD169-DTR, CD11c-DTR, and IFNAR KO mice at day 10 p.v. (n = 10). **(D)** Histogram showing the capture of BNT162b2 vaccine at 12 h p.v. (left) and the expression of coronavirus spike protein at 48 h p.v. (right) by pDCs, CD11b^+^ DCs, and CD11b^−^ DCs in the pLN. (n = 5). **(E)** Confocal micrograph showing a transverse section of a pLN (left) and magnifications of the T cell zone (TCZ, right) indicating vaccine capture and spike expression in PDCA^+^ pDCs at 12 h p.v. Colours indicate CD21/35^+^ follicles (blue), CX3XR1^+^ cells (green), PDCA^+^ pDCs (cyan), BNT162b2 vaccine (white), and spike protein (red). F stands for B cell follicle. **(F)** Quantification of spike^+^ pDCs in the different areas of the pLN at 12 h p.v. **(G)** Violin plots showing the ratio of IFN-β (left) and IFN-γ (right) expression in the lymph of CD169-DTR, CD11c-DTR, aPDCA, and IFNAR KO mice compared to WT mice at 12 h p.v. (Left graph: n = 20 for WT group, n = 15 for CD169-DTR, CD11c-DTR and aPDCA groups, n = 24 for IFNAR KO group; Right graph: n = 20 for WT group, n = 9 for CD169-DTR, CD11c-DTR/GFP groups, n = 16 for aPDCA group, n = 24 for IFNAR KO group). Differences not reaching statistical significance are represented by the absence of asterisks. **(H)** AUC for anti-SARS-CoV-2 RBD IgG in WT and aPDCA-treated mice at day 10 p.v. (n = 6). **(I)** Flow cytometric analysis showing the ratio of SARS-CoV-2 RBD specific memory B cells in aPDCA mice compared to WT at day 10 p.v. (n = 4). **(B and C, H)** The graph is representative of three independent experiments. Data are presented as mean ± SD. Two-way ANOVA **(A)** and One-way ANOVA **(B–D, FandG)** followed by Bonferroni correction for multiple comparisons. Student’s t-tests **(HandI)** (∗p < 0.05, ∗∗p < 0.01, ∗∗∗p < 0.001 ∗∗∗∗p < 0.0001).

**Fig. 3:**
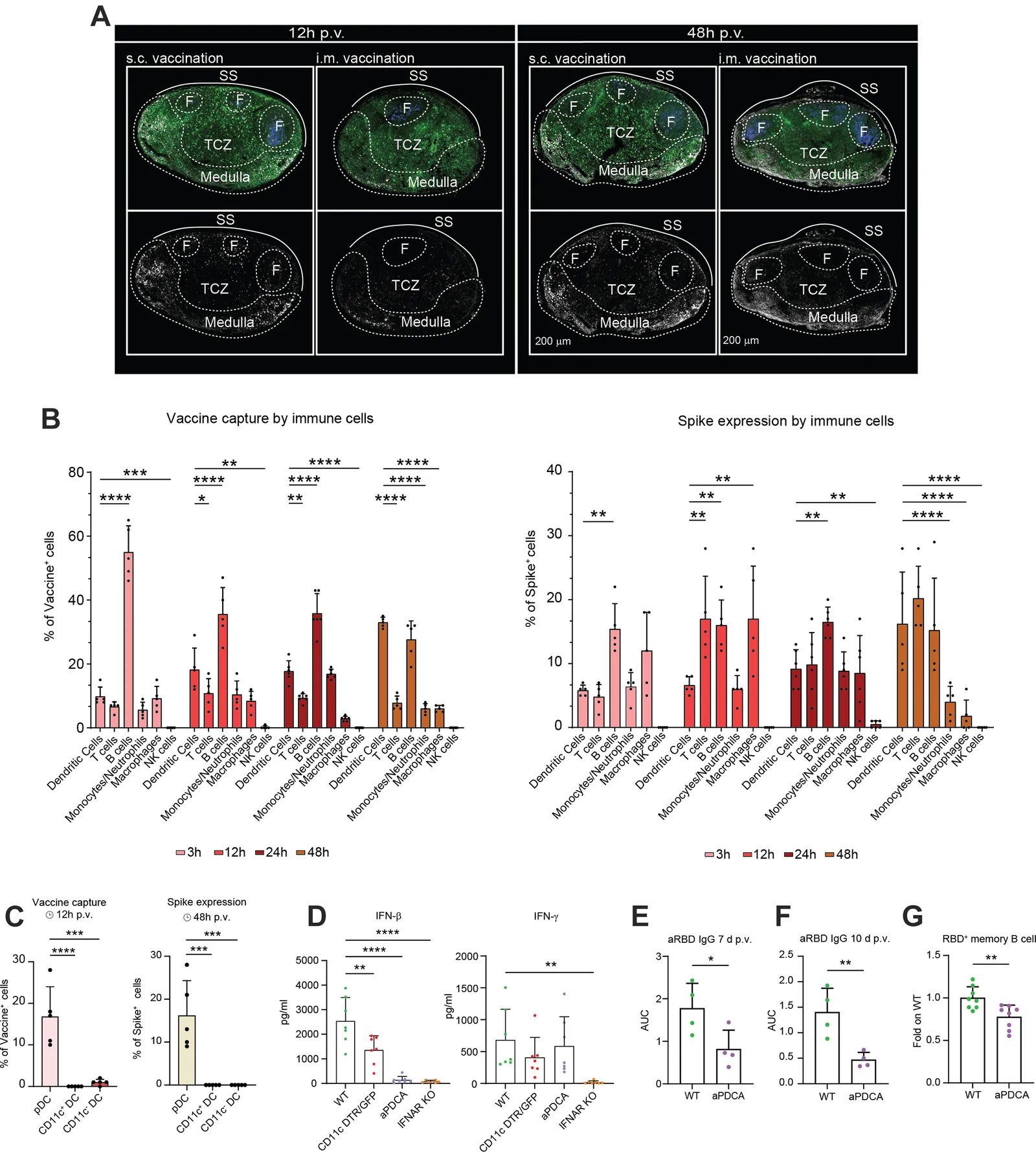
**pDCs contribute to triggering the immune response following intramuscular BNT162b2 vaccination. (A)** Representative confocal micrograph showing a transverse section of a pLN indicating vaccine distribution at 12 h (above) and 48 h (belove) p.v. in s.c. or i.m. vaccinated mice. Colours indicate CD21/35^+^ follicles (blue), CX3XR1^+^ cells (green), BNT162b2 vaccine (white). The micrographs show independent representative sections from different animals used under respective experimental conditions. F stands for B cell follicle; SS stands for subcapsular sinus; TCZ stands for T cell zone. **(B)** Histogram showing the frequency of immune cells positive for the BNT162b2 vaccine (left) and the coronavirus spike protein (right) in the pLN at different time points post intramuscular vaccination (p.i.v.) (n = 5 for each group). **(C)** Histogram showing the capture of BNT162b2 vaccine at 12 h p.i.v. (left) and the expression of coronavirus spike protein at 48 h p.i.v. (right) by pDCs, CD11b^+^ DCs, and CD11b^−^ DCs in the pLN. (n = 5). **(D)** Histogram showing the concentration of IFN-β (left) and IFN-γ (right) in the lymph of CD11c-DTR, aPDCA, and IFNAR KO mice compared to WT mice at 12 h p.i.v (n = 7 for each group). Differences not reaching statistical significance are represented by the absence of asterisks. **(E)** AUC for systemic anti-SARS-CoV-2 RBD IgM for WT and aPDCA-treated mice at day 7 p.i.v (n = 4 for each group). **(F)** AUC for systemic anti-SARS-CoV-2 RBD IgG for WT and aPDCA-treated mice at day 10 p.i.v (n = 4 for each group). **(G)** Flow cytometric analysis showing the ratio of SARS-CoV-2 RBD specific memory B cells in the pLN of aPDCA mice compared to WT at day 10 p.i.v (n = 8 for each group). Data are presented as mean ± SD. Student’s t-test **(E–G)**. One-way ANOVA **(C, D)**, and Two-way ANOVA **(B)** followed by Bonferroni correction for multiple comparisons. (∗p < 0.05, ∗∗p < 0.01, ∗∗∗p < 0.001 ∗∗∗∗p < 0.0001).

To further characterise the immune cell types involved in vaccine uptake and antigen expression, we performed a time course analysis in the pLN. Our study revealed that, among the cells capturing the vaccine, T and B cells were the most abundant (Fig. 2A, left panel). Instead, analysing the cells expressing the spike protein, the APC, particularly dendritic cells (DCs), represented the predominant cell population (Fig. 2A, right panel). To determine the specific role of the different phagocytic populations, we analysed the antibody response in vaccinated animals depleted of conventional DCs (cDCs) (*CD11c-DTR*) or macrophages (*CD169-DTR*). Given the critical role of the type I interferon (IFN) pathway in triggering an efficient humoural response to vaccines,^34^ we also included a group of mice lacking the IFN type I receptor (IFNAR). We observed that DC depletion, but not macrophage depletion, was associated with a significant reduction in the systemic (Fig. 2B) and the local (Fig. 2C) antibody production, like that observed in *IFNAR knockout (KO)* mice. To further explore the specific contribution of different DC subsets to vaccine-induced immunity, we studied vaccine capture at 12 h p.v. and spike expression at 48 h p.v. in the three major groups of DCs (B220^+^ PDCA^+^ pDC, CD11b^+^ cDCs and CD11b^−^ cDCs). Notably, pDCs exhibited the highest percentage of cells positive for the vaccine and the spike protein at the evaluated time points (Fig. 2D). To confirm these results using a spatial imaging approach, we performed confocal microscopy analysis of sections at 12 h p.v., observing a pronounced accumulation of vaccine^+^ spike^+^ pDCs in the medulla and the T cell zone of the draining LN (Fig. 2E and F). To further define the role of pDCs in the response to the vaccine, we first established an *in vivo* model of pDC-depleted mice based on previously published protocols,^21^^,^^22^ with the depletion regimen adapted according to pDC recovery kinetics (Supplementary Fig. 2A). Then, we assessed the levels of interferon response in mice depleted of pDCs (*aPDCA*), cDCs (*CD11c-DTR*), macrophages (*CD169-DTR*), and in *IFNAR KO* mice at 12 h p.v. Interestingly, pDC-depleted mice, but not cDC- or macrophage-depleted mice, showed a reduction in IFN-β and IFN-γ protein levels, comparable to those observed in *IFNAR KO* mice (Fig. 2G). Furthermore, mice depleted of pDCs exhibited a significant reduction in the anti-SARS-CoV-2 RBD IgG levels following vaccination (Fig. 2H). In addition, we observed a reduced production of anti-SARS-CoV-2 RBD IgM on day 7 (Supplementary Fig. 2B), as well as a lower generation of anti-SARS-CoV-2 RBD IgG and RBD-specific memory B cells on day 10 p.v. (Fig. 2I). Despite the reduced antibody titre, the neutralising capacity was not affected in the pDC-depleted mice compared to the WT group (Supplementary Fig. 2C). To strengthen the clinical relevance of our findings, we repeated the key experiments using i.m. vaccination with the same vaccine dose. These data show that following i.m. administration, the vaccine similarly accumulates within the pLN, preferentially localising to the medullary region, albeit with a modest delay compared to s.c. administration (Fig. 3A). Then, we analysed the vaccine capture and spike expression by immune cells (Fig. 3B), and, more specifically, by the major DC subtypes (Fig. 3C). Despite higher spike protein expression by T and B cells following i.m. vaccination (Fig. 3B), pDCs remained the predominant APCs showing the highest levels of the vaccine capture and viral antigen expression (Fig. 3C). Moreover, type I IFN response closely resembled the one observed after s.c. vaccination, with comparable IFN-β reduction in aPDCA vs. WT mice (Fig. 3D, left graph). In contrast, the reduction in IFN-γ observed following s.c. vaccination was not detected in the i.m. setting (Fig. 3D, right graph), a difference that is likely a consequence of the increased frequency of spike^+^ T cells, which are known to be a possible source of IFN type II. Finally, humoural immune responses in aPDCA-treated mice following i.m. vaccination (Fig. 3E–G) mirrored those induced by s.c. vaccination.

### Single-cell transcriptomic analysis predicts an interaction between pDCs and CD8^+^ T cells

**Fig. 4:**
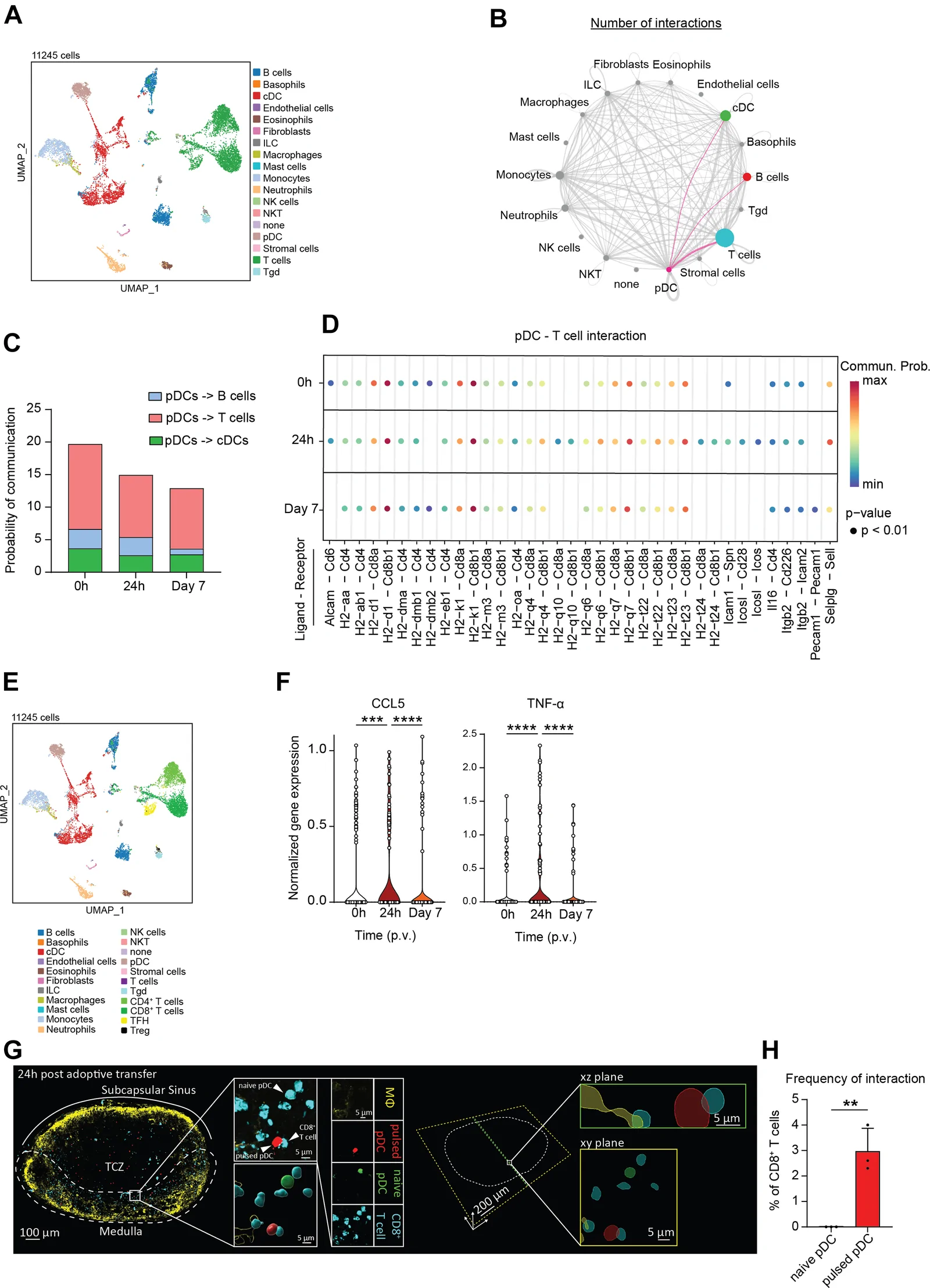
**Cell-cell interaction analysis predicts communication between pDCs and CD8+ T cells following vaccination. (A)** UMAP of cell types clustered by single-cell transcriptional analysis of 11,245 cells after quality control. (n = 1 for each group). cDC, classical dendritic cell; ILC, innate lymphoid cell; NK, natural killer cell; NKT, natural killer T cell; pDC, plasmacytoid dendritic cell; Tgd, γδ T cell. **(B)** Circle plot showing the intercellular communication between the major immune cell types in the dLN after vaccination. **(C)** Histogram showing the probability of communication between pDCs with T cells, B cells, or cDCs at 0 h, 24 h, and 7 days p.v. **(D)** Comparison of the significant ligand-receptor pairs between pDCs and T cells in the dLN p.v. Dot colour reflects communication probabilities, and dot size represents computed p-values. Space indicates that the communication probability is zero. p-values are calculated from one-sided permutation test. **(E)** UMAP of cell types clustered by single-cell transcriptional analysis of 11,245 cells after quality control. cDC, classical dendritic cell; ILC, innate lymphoid cell; NK, natural killer cell; NKT, natural killer T cell; pDC, plasmacytoid dendritic cell; Tgd, gd T cell; TFH, follicular T helper cell; Treg, regulatory T cell. **(F)** Truncated violin plots showing the gene expression of CCL5 (left) and TNF-α (right) expressed by pDCs at single-cell levels at 0 h, 24 h, and 7 days p.v. Each dot represents a single cell. **(G)** A representative confocal micrograph shows a transversal section of the pLN (left) and a magnification view showing the interaction between a pulsed pDCs and a CD8^+^ T cells (centre). A representative mask shows the overlapping of the pulsed pDCs and CD8^+^ T cells signals (Right). Colours indicate CD169^+^ macrophages (MΦ) (yellow), vaccine-pulsed pDCs (red), naïve pDCs (green and cyan), and CD8^+^ T cells (cyan). **(H)** Histogram showing the frequency of CD8^+^ T cells interacting with naïve pDCs or *in vitro* pulsed pDCs 24 h post adoptive transfer. Data are presented as mean ± SD. One-way ANOVA followed by Bonferroni correction for multiple comparisons **(F)**. Student’s t-tests **(H)** (∗p < 0.05, ∗∗p < 0.01, ∗∗∗p < 0.001 ∗∗∗∗p < 0.0001).

To explore potential pDC mediated cell-cell interaction, we analysed a previously published single-cell sequencing dataset generated from the dLNs of mice following intramuscular immunisation with the BNT162b2 vaccine.^16^ With the CellChat analysis tool,^33^ we quantitatively characterised the intracellular communication network. This analysis was performed at 0 h, 24 h, and 7 days p.v. Using dimensionality reduction via uniform manifold approximation and projection (UMAP) and graph-based clustering, we identified 18 cell clusters encompassing all major innate and adaptive immune cell types (Fig. 4A). Subsequent analyses enabled visualisation of intercellular communication dynamics over time. Our investigation focused on the direct interaction of pDCs with B cells and their potential role in T-dependent antibody response, mainly through interactions with T cells or cDCs. The circle plot, which depicts cell numbers by the circle size and communication intensity by the line thickness, reveals a stronger probability of interaction between pDCs and T cells (Fig. 4B). To further examine this, we evaluated the probability of communication between pDCs and B cells, T cells, or cDCs over time. Notably, we identified a higher communication probability between pDCs and T cells compared to B cells or cDCs (Fig. 4C). Ligand-receptor (L-R) analysis further identified unique interactions at 24 h p.v., including complexes such as H2-q10—CD8a, H2-q10—CD8b1, H2-t24—CD8a, H2-t24—CD8b1, Icosl—CD28, Icosl—Icos. These findings suggest a communication between pDCs and CD8^+^ T cells (Fig. 4D).

To determine which T cell subset was predominantly targeted by pDCs, we re-annotated the cell clusters to distinguish CD4^+^ and CD8^+^ T cells as separate clusters (Fig. 4E). Moreover, separate L-R analyses were then performed for each subset (Supplementary Fig. 4A). Both CD4^+^ and CD8^+^ T cells showed significant communication with pDCs across all timepoints, reflecting the broad immunological role of pDCs in orchestrating the adaptive response following vaccination. However, the pDC–CD8^+^ T cell interaction profile appeared particularly rich, displaying a higher number of significant L-R pairs and greater overall communication probabilities compared to the CD4^+^ subset, especially at 24 h p.v. These CD8-exclusive interactions, combined with the broader and more dynamic L-R repertoire observed in the CD8^+^ subset, prompted us to focus our subsequent analyses on the pDC–CD8^+^ T cell axis (Supplementary Fig. 4A, right graph).

To validate the L-R interactions, we assessed the expression levels of the relevant L-R genes in the re-annotated clusters (Fig. 4E). At 24 h p.v., we observed increased Icosl, H2-q10, and H2-t24 expression in pDCs compared to baseline (0 h) and day 7. Additionally, pDCs activation was further corroborated by the upregulation of CD69 and the mRNA sensing gene Ifih1 (Supplementary Fig. 4B). Concurrently, we found that CD8^+^ T cells displayed an increased expression of Icos and CD28, as well as a significant expression of the activation markers CD69 and Gzmb at 24 h p.v. (Supplementary Fig. 4C).

To support the predicted CD8^+^ T cell–pDC interaction, we also analysed the expression of CCL5 and TNF-α, confirming an upregulated gene expression at 24 h p.v. compared to both 0 h and day 7 (Fig. 4F). To experimentally confirm these results, we performed a new set of experiments, evaluating the protein level of CCL5 and TNF-α in vaccinated aPDCA-treated and *IFNAR KO* mice compared to WT mice at 12 h p.v. As expected, depletion of pDCs or loss of IFNAR signalling markedly reduced the levels of CCL5 and TNF-α compared to the control groups (Supplementary Fig. 4D). To support the predicted interactions previously identified, we performed adoptive transfer experiments with fluorescently labelled naïve CD8^+^ T cells (cyan), naïve pDCs (cyan and green), and *in vitro* pulsed pDCs (red) in C57BL/6 mice. After 24 h, the pLNs were collected and analysed by confocal microscopy. The results revealed a significant increase in the interactions between vaccine-pulsed pDCs and CD8^+^ T cells. In contrast, naïve pDCs failed to establish such interactions (Fig. 4G and H). These findings suggest a potential role of spike^+^ pDCs in directly engaging and activating CD8^+^ T cells within the pLN, thereby facilitating their functional differentiation post-vaccination.

### Spike^+^ pDCs engage with CD8^+^ T cells, contributing to their activation in the pLN

**Fig. 5:**
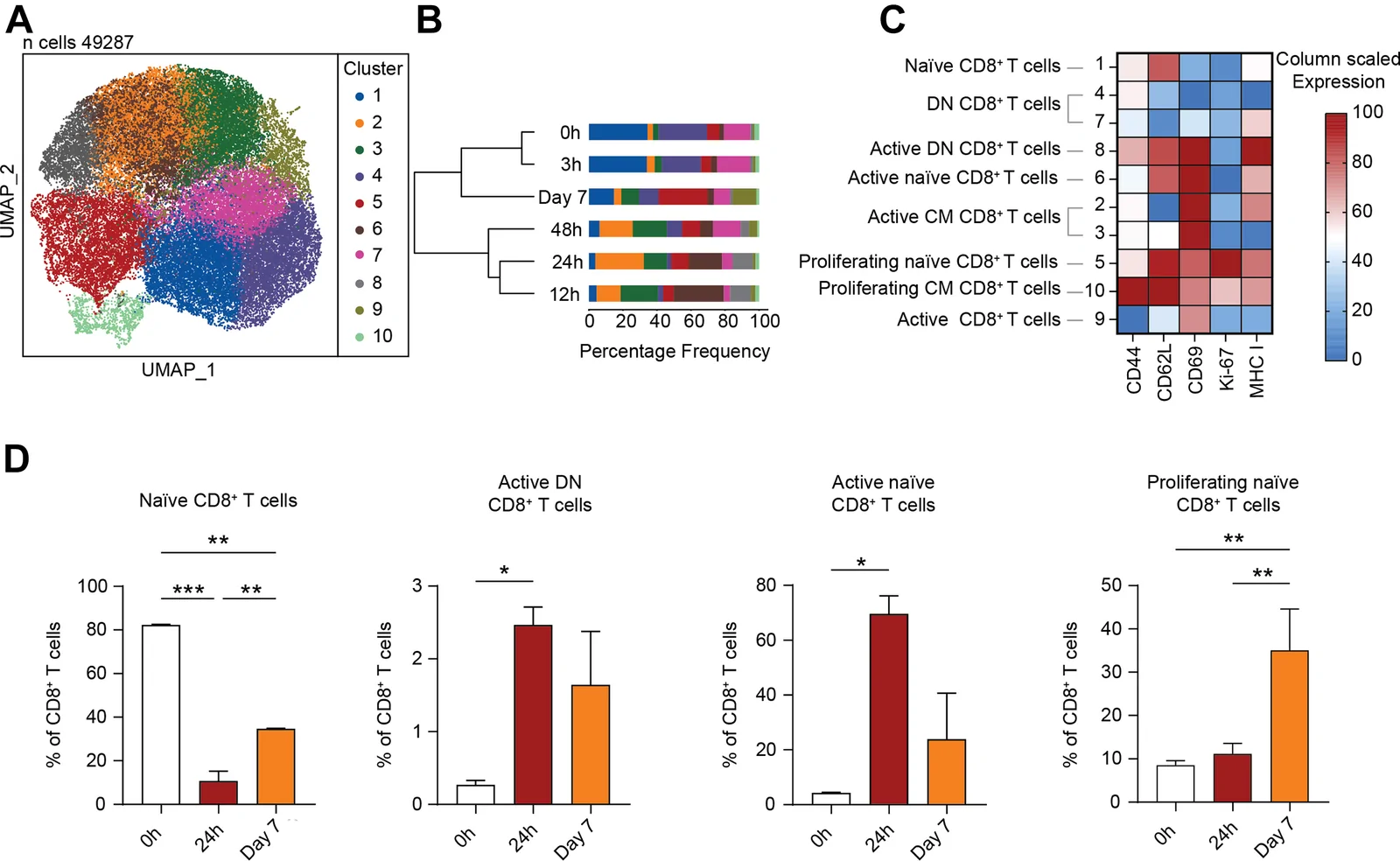
**T cell activation induced by BNT162b2 vaccine in the pLN. (A)** UMAP plot showing the clusters of CD8^+^ T cell obtained from flow cytometrical analysis of 49,287 cells p.v. **(B)** Stacked-bar graph showing the percentages of CD8^+^ T cells Phenograph clusters. **(C)** Heatmap showing the column-scaled expression of markers (columns) in discrete CD8^+^ T cell Phenograph clusters (rows). **(D)** Histogram showing the main CD8^+^ T cell subsets at 0 h, 24 h, or 7 days p.v. in the pLN (n = 3 for each group). Differences not reaching statistical significance are represented by the absence of asterisks. Data are presented as mean ± SD. One-way ANOVA followed by Bonferroni correction for multiple comparisons (∗p < 0.05, ∗∗p < 0.01, ∗∗∗p < 0.001 ∗∗∗∗p < 0.0001).

To investigate the phenotypic changes in the CD8^+^ T cell population following vaccination, we performed cluster analysis using the Phenograph algorithm, which identified 10 distinct CD8^+^ T cell subsets (Fig. 5A), exhibiting dynamic changes over time (Fig. 5B; Supplementary Fig. 5A). A heatmap displaying the normalised marker expression of CD44, CD62L, Ki-67, and MHC I across clusters allowed us to determine cluster identities (Fig. 5C). Quantitative analysis of CD8^+^ T cells frequencies revealed a significant decline in naïve CD8^+^ T cells at 24 h p.v., coinciding with an increase in active double negative (DN) and active naïve CD8^+^ T cells at the same time point (Fig. 5D). By day 7 p.v., this transition culminated in a notable expansion of proliferating naïve CD8^+^ T cells (Fig. 5D, Supplementary Fig. 5B). Conversely, other clusters identified by the UMAP analysis did not exhibit statistically significant differences over time (Supplementary Fig. 5C). Overall, these results describe the activated state of CD8^+^ T cells post-vaccination.

### Spike^+^ pDCs are involved in the antigen-specific memory T cell formation following vaccination

Several studies have confirmed the induction of antigen-specific T cells after BNT162b2 vaccination.^35^^,^^36^ Additionally, type I IFNs have been shown to directly stimulate CD8^+^ T cells, promoting clonal expansion and memory formation.^35^ Based on these findings, we investigated whether pDCs depletion impairs the T-cell response to vaccination.

**Fig. 6:**
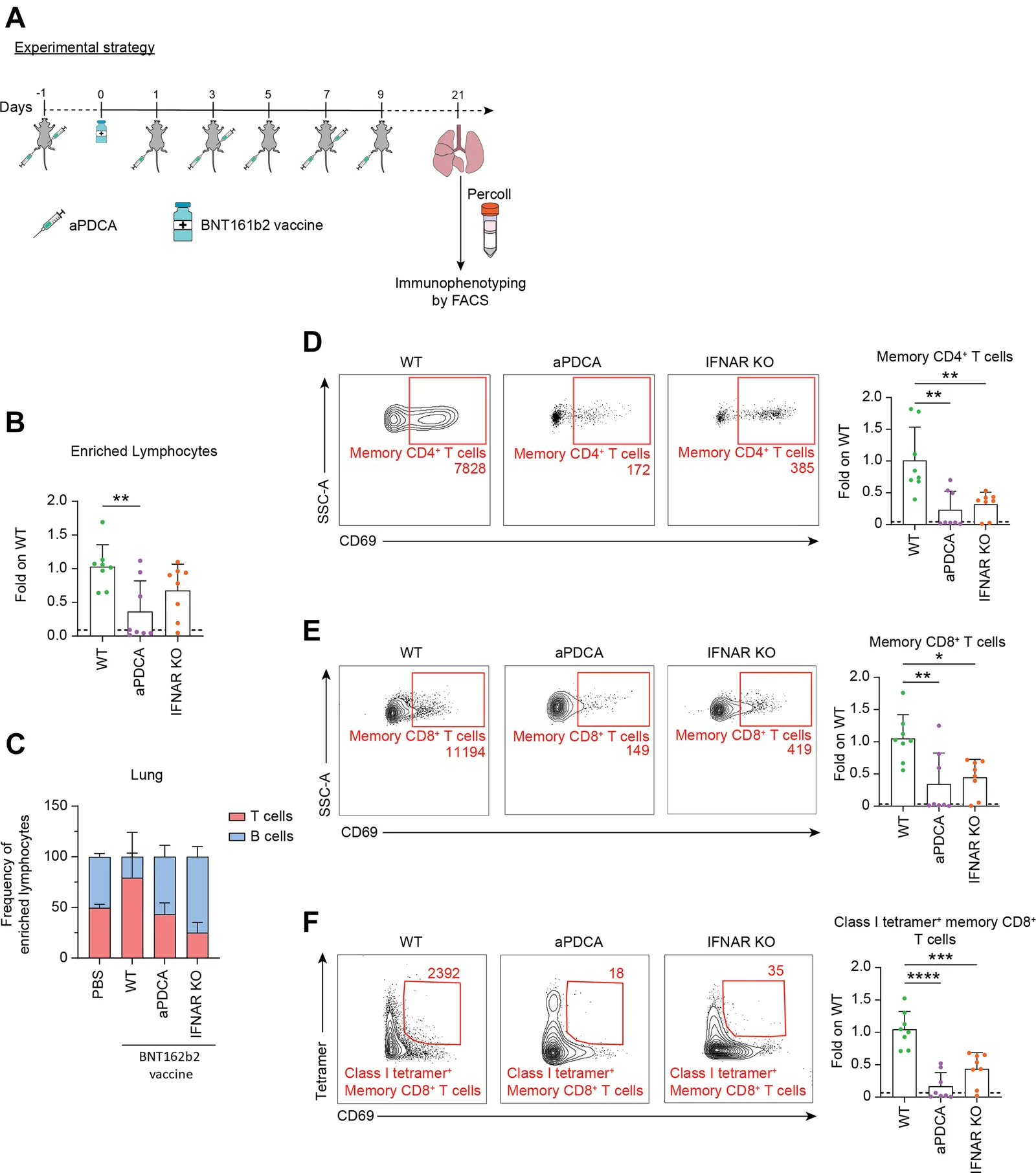
**Spike + pDCs are involved in BNT162b2 vaccine-induced memory CD8+ T cells formation. (A)** Experimental strategy. **(B)** Histogram showing CD45^+^ cell counts in the Percoll-enriched lymphocyte fraction from the lungs of PBS vaccinated (dashed line) or BNT162b2 vaccinated WT, aPDCA-treated, and IFNAR KO mice, measured on day 21 p.v. Differences not reaching statistical significance are represented by the absence of asterisks. **(C)** Stacked bar graph showing the frequency of B and T cells in the Percoll-enriched lymphocyte fraction from the lungs of PBS vaccinated or BNT162b2 vaccinated WT, aPDCA-treated, and IFNAR KO mice, measured on day 21 p.v. **(D)** Flow cytometry contour plot (above) from a representative experiment out of 3 and histogram (below) showing memory CD4^+^ T cells count in the Percoll-enriched lymphocyte fraction from the lungs of PBS vaccinated (dashed line) or BNT162b2 vaccinated WT, aPDCA and IFNAR KO mice, measured on day 21 p.v. **(E)** Flow cytometry contour plot (above) from a representative experiment out of 3 and histogram (below) showing memory CD8^+^ T cells count in the Percoll-enriched lymphocyte fraction from the lungs of PBS vaccinated (dashed line) or BNT162b2 vaccinated WT, aPDCA-treated and IFNAR KO mice, measured on day 21 p.v. **(F)** Flow cytometry contour plot (above) from a representative experiment out of 3 and histogram (below) showing Class I tetramer^+^ memory CD8^+^ T cells count in the Percoll-enriched lymphocyte fraction from the lungs of PBS vaccinated (dashed line) or BNT162b2 vaccinated WT, aPDCA-treated and IFNAR KO mice, measured on day 21 p.v. **(B–F)** The experimental groups were made of n = 3 for PBS, n = 8 for all the other groups. Data are presented as mean ± SD. One-way ANOVA followed by Bonferroni correction for multiple comparisons (∗p < 0.05, ∗∗p < 0.01, ∗∗∗p < 0.001 ∗∗∗∗p < 0.0001).

To address this point, we analysed the lymphocyte infiltration in the lungs of vaccinated WT, aPDCA-treated, and *IFNAR KO* mice (Fig. 6A). Both aPDCA-treated and *IFNAR* KO mice exhibited a marked reduction in lymphocyte infiltration, comparable to non-vaccinated controls (Fig. 6B). Further analysis of T and B cell ratios revealed that only vaccinated WT mice, but not aPDCA-treated or *IFNAR KO* mice, showed T cell enrichment (Fig. 6C). Supporting our hypothesis, the depletion of pDCs or the absence of IFNAR resulted in a significant decrease in the overall memory CD4^+^ and CD8^+^ T cell populations (Fig. 6D and E). Notably, the spike-specific CD8^+^ T cell subset experienced the most pronounced reduction, underscoring the critical role of pDCs in promoting T cell memory formation (Fig. 6F).

## Discussion

This study intended to elucidate the mechanisms by which the BNT162b2 NP-based vaccine elicits effective humoural and cellular immunity against SARS-CoV-2.

While previous studies described vaccine arrival in the LN,^37^ it remained unclear whether this transport was associated with migrating APCs or occurred via lymphatic drainage.^16^^,^^38^ Here, we demonstrated that soon following injection, the BNT162b2 vaccine drains via the lymphatic system. Indeed, while the immune cells expressing the viral antigen require one to four days to migrate from the injection site to the dLN,^39^^,^^40^ the inflammatory response is initiated in this organ already at 3 h post-vaccination. This rapid activation highlights the role of LN-resident leukocytes in the early uptake and presentation of the antigen. However, this process might be influenced afterward by the migration of immune cells from the injection site. The impact of these events on the immune response to the vaccine needs to be further evaluated. Notably, the size and the surface composition of the drug carrier can significantly affect the pharmacokinetics and biodistribution properties of the associated small molecules.^41^ For example, Cordeiro and colleagues showed how different sizes and surface properties of polymeric nanocapsules influence their ability to target specific regions of the LN, consequently modulating immune cell engagement.^42^ Based on these studies, we could expect differences in the capture and distribution of other commercially available coronavirus vaccines in the dLN.^43^ Indeed, we observed a preferential accumulation of the BNT162b2 vaccine in the interfollicular and medullary regions of the LN. This is most likely due to the convergence of a high number of medullary sinuses, increasing the draining surface, and the presence of different types of phagocytic populations,^44^ responsible for the rapid capture of the vaccine and the expression of the spike protein. The distribution of the BNT162b2 vaccine was consistent with previous observations that identified the interfollicular region and the medulla as the key targets not only for inactivated vaccines (as the influenza type I vaccine), which are often considered highly effective natural vehicles for nucleic acid delivery, but also for polymeric nanoparticles.^9^^,^^45^^,^^46^

Consistent with previous studies highlighting the role of APCs during mRNA vaccination,^16^ we observed that pDCs exhibited the highest vaccine uptake and spike protein expression following vaccination. Although the capacity of pDCs to capture nanoparticles has been demonstrated *in vitro*,^47^ their involvement *in vivo*, especially in the context of vaccination, is still poorly understood. Brewitz and colleagues studied the localisation of pDCs at steady state in the LN, finding a higher abundance of these cells in the interfollicular, paracortical, and medullary areas, preferentially associated with high endothelial venules. These authors also found that pDCs migrate towards the medulla and the T cell zone upon vaccination.^48^ In our model, the vaccine accumulates in the same regions where pDCs are located, which could explain their higher capture compared to other immune cells. In addition, different studies have shown that viral RNA can be transferred between infected cells and pDCs through exosomes.^49^^,^^50^ Therefore, the expression of the spike protein observed in the pDCs could be the result of the direct capture of the vaccine by the pDCs, but also the result of the transfer of the RNA or the proteins expressed by other cells located in the same area.

By analysing the contribution of pDCs in establishing a sustained adaptive immune response following vaccination, we found that the absence of this population significantly compromised the development of the adaptive response, affecting both the humoural and the cell-mediated response. Regarding the humoural response, it is not completely understood how B cells encounter their antigen. Different mechanisms have been proposed for antigen presentation to B cells upon infection or vaccination. In case of small antigens (<70 kDa) their capture occurs following a direct drainage via the conduit system located in the B follicles.^51^ Conversely, larger antigens are captured by macrophages or cDCs in the subcapsular sinus and medullary regions and are transferred to the B cells.^9^ Taking this into account we hypothesise that pDCs, as well as classical DCs, might relocate towards the B cell follicle and present the newly express antigen directly to the B cells or transfer it to the FDC network.

Furthermore, we, and others, have previously demonstrated the critical role of the type I IFN signalling in the development of a proper antibody response upon immunisation.^20^^,^^34^^,^^52^ In this work, we proposed an additional scenario in which the contribution of pDCs to the humoural response is associated with their role in the initiation of the type I IFN response that follows vaccination. Here we identified pDCs as contributors to the IFN-β production in the dLN. Furthermore, Li and colleagues demonstrated that the mRNA vaccine is sensed by LN-resident cells by engaging the MDA5-IFNAR1 signalling pathway.^16^ In addition, activated macrophages were shown to enhance type I IFN secretion by pDCs in the context of SARS-CoV-2 infection.^53^ Supporting the involvement of pDCs in the sensing of the vaccine, we observed a strong upregulation of the related gene (*Ifih1*) following vaccination. This suggests that pDCs act as early sentinels for mRNA vaccines, integrating innate immune sensing with downstream adaptive immune activation. Taken together, these findings suggest the involvement of the pDCs in orchestrating early events that support the development of effective humoural immunity.

Interestingly, despite the reduction in anti-SARS-CoV-2 RBD IgG and IgM observed in pDC-depleted mice, neutralising antibody titres were comparable to those WT vaccinated animals. This suggests that pDCs are not strictly required for the early generation of neutralising activity, which may depend on a limited pool of high-affinity antibodies or on compensatory antigen-presenting pathways involving B cells and other APC subsets. Although this study primarily focuses on early immunological events following mRNA vaccination, longitudinal analyses will be required to determine whether pDCs influence the durability, affinity maturation, and class distribution of neutralising antibodies. Such studies will be essential to clarify whether pDCs predominantly shape the magnitude and long-term persistence of humoural immunity rather than its initial neutralisation potency.

Along with B cells, type I IFN contributes to different immunological processes such as the T cell memory response against viruses^54^ or the migration of pDCs within the lymph node following viral infection.^55^^,^^56^ In addition to that, a previous study showed that the MDA5-IFNAR1 signalling pathway is also critical for CD8^+^ T cell response induced by the BNT162b2 vaccine.^16^ We extended these findings to show that spike^+^ pDCs were able to establish direct interactions with CD8^+^ T cells, suggesting their potential role in directly engaging and activating T cells within the dLN. Strengthening this hypothesis, we observed a significant loss of antigen-specific memory CD8^+^ T after immunisation in mice lacking pDCs. Interestingly, similar pDCs–CD8^+^ T cell interactions have been described during viral infections.^50^^,^^57^ For example, Mouriès and colleagues demonstrated the capacity of pDCs to cross-present exogenous antigen to naïve T cells, resulting in their activation and maturation.^21^ This direct interaction provides mechanistic evidence that pDCs may function beyond their classical role in type I IFN production to act as unconventional APCs during vaccination. The growing interest in pDCs in the cancer immunology field further supports their potential as targets for optimising future vaccines.^46^^,^^47^^,^^58^, ^59^, ^60^, ^61^ For example, Butkovich and colleagues showed how RNA-loaded nanoparticles can efficiently activate pDCs, improving the tumoral antigen presentation by cDCs to CD8^+^ T cells.^47^

Several limitations of our study should be acknowledged. First, our experiments were performed using mouse models, which, although highly informative for mechanistic studies, may not fully reflect the complexity of human lymphoid architecture or vaccine response. Second, although BNT162b2 vaccine is administered intramuscularly in humans, our initial experiments employed subcutaneous injection to enable precise spatiotemporal analysis of vaccine drainage and immune cell interactions within a defined draining LN. While this approach is widely used in murine immunology and intravital imaging,^61^^,^^62^ it may influence the magnitude and quality of immune responses, as s.c. and i.m. delivery have been reported to shape T cell immunity in mice differentially.^63^ To address this point, we have repeated the key experiments using i.m. vaccination and we observed a similar outcome in both models (Fig. 3A-G).

In addition, our analysis of vaccine-induced T cell responses focused on spike-specific CD8^+^ T cells rather than spike-specific CD4^+^ T cells. This decision was guided by several studies highlighting the critical role of spike-specific CD8^+^ T cells induced by BNT162b2 vaccination in antiviral protection and long-term memory. In particular, BNT162b2 has been shown to elicit robust spike-specific CD8^+^ responses that depend on MDA5–IFNAR1 signalling in mice,^16^^,^^64^ as well as to preferentially induce spike-specific CD8^+^ memory clonotypes and effector-memory or tissue resident-like CD8^+^ T cells in humans.^19^ Consistent with this literature, despite the single-cell transcriptomic and ligand–receptor analyses predicted a direct interaction of pDCs with both CD4^+^ and CD8^+^ T cells, the pDC–CD8^+^ T cell communication profile appeared particularly rich with a broader and more dynamic L-R repertoire. Moreover, to confirm the results, we subsequently validated experimentally *in vivo*. Indeed, since statistical inference is limited in the absence of biological replicates, this may increase the risk of false positives, so we did not base any mechanistic conclusions exclusively on these single-cell statistics. Instead, we validated the main results through multiplex cytokine measurements. Although direct pDC-CD8^+^ T cell contacts were detected, quantifying the spatial distribution and relative migratory dynamics of pDCs before and after encountering the vaccine remains to be established. In addition, given the important role of CD4^+^ T cells in shaping both humoural and cellular immunity, future studies will be required to determine whether pDCs also influence CD4^+^ T cell priming, differentiation, and memory formation following mRNA vaccination.

Third, while we identify pDCs as major early targets of the vaccine and as an important source of type I IFN, our data do not fully resolve whether the spike expression detected in pDCs derives from direct vaccine uptake, transfer of mRNA or protein from neighbouring cells, or a combination of these mechanism, nor do they exclude important contributions from other antigen-presenting cell subsets (as cDCs or B cells). Indeed, given the complexity of this novel vaccination strategy, the involvement of additional cell types capable of vaccine uptake and viral antigen expression is likely to contribute to the residual response observed despite pDC depletion. Finally, our work focused on early events following a single vaccine dose, and therefore does not address how pDC-mediated mechanisms may differ after booster immunisations or during recall responses.

In conclusion, our results provide evidence regarding the *in vivo* mechanisms underlying the immune response to the BNT162b2 vaccine and suggest a contribution of pDCs in mediating vaccine-induced immunity. Our findings contribute to a better understanding of nanoparticle-based vaccine-induced immune response and may help inform future efforts aimed at improving vaccine efficacy in infectious and cancer immunotherapies.

## Contributors

**C.P.:** Conceptualisation, data curation, formal analysis, investigation, visualisation, methodology, writing–original draft, project administration, writing–review and editing. **I.L.:** Conceptualisation, data curation, formal analysis, funding acquisition, investigation, visualisation, methodology, project administration. **T.V.**: Investigation, methodology. **A.C.**: Investigation, methodology. **K.C.**: Investigation. **L.C.**: Single-cell data curation and verification, formal analysis, visualisation, methodology. **S.G.M.**: Single-cell data curation and verification, formal analysis, visualisation, methodology. **G.B.**: investigation. **M.B.**: Resources, investigation, methodology. **N.K.:** Resources, investigation, methodology. **A.P.**: Investigation, methodology, formal analysis. **L.R.**: investigation. **R.S.T.**: Investigation, methodology. **C.B.**: Resources, investigation, methodology. **D.F.L.**: Conceptualisation, discussion, supervision. **S.F.G.**: Conceptualisation, resources, data curation, supervision, funding acquisition, writing original draft, project administration, writing–review and editing. **C.P. and S.F.G.** directly accessed and verified the underlying data reported in the manuscript. All authors reviewed and edited the manuscript, approved the final version, and agreed to be accountable for all aspects of the work.

## Data sharing statement

Data supporting the findings of this study will be made available upon reasonable request, beginning at publication and with no end date. Proposals should be directed to santiago.gonzalez@irb.usi.ch.

## Declaration of interests

The authors declare that they have no known competing financial interests or personal relationships that could have appeared to influence the work reported in this paper.
