# Supplementary material for: Role of plasmacytoid dendritic cells in humoural and CD8+ T-cell memory responses following BNT162b2 mRNA vaccination: a preclinical mouse study
Source: eBioMedicine. 2026 Aug 21;131:106432. doi: 10.1016/j.ebiom.2026.106432 (PMC13524885; doi:10.1016/j.ebiom.2026.106432)
Supplement: Supplementary file 1 [file mmc1.docx]

**Supporting Information for**

**Role of Plasmacytoid Dendritic Cells in Humoral and CD8⁺ T-Cell Memory Responses Following BNT162b2 mRNA Vaccination: a Preclinical Mouse Study**

*Chiara Pizzichetti^1,2Ψ^, Irene Latino^1Ψ^, Tommaso Virgilio^1*^, Arianna Capucetti^1*^, Kamil Chahine^1, 2^, Luciano Cascione^3^, Simone G. Moro^1^, Giorgia Bilato^10, 11^, Melanie Brügger^4, 5^, Nedim Kozarac^2, 4, 5^, Alain Pulfer^1^, Louis Renner^1^, Roshan S. Thakur^1^, Charaf Benarafa^4, 5, 6^, Daniel F. Legler^7,8,9^, Santiago F. González^1&^.*

*^1^ Università della Svizzera italiana, Faculty of Biomedical Sciences, Institute for Research in Biomedicine, Bellinzona, Switzerland.*

*^2^ Graduate School for Cellular and Biomedical Sciences, University of Bern, Bern, Switzerland.*

*^3^ Università della Svizzera italiana, Faculty of Biomedical Sciences, Institute of Oncology Research, Bellinzona, Switzerland.*

*^4^ Institute of Virology and Immunology, Bern and Mittelhäusern, Bern, Switzerland.*

*^5^ Department of Infectious Diseases and Pathobiology, Vetsuisse Faculty, University of Bern, Bern, Switzerland.*

*^6^ Multidisciplinary Center for Infectious Diseases, University of Bern, Bern, Switzerland.*

*^7^ Institute of Cell Biology and Immunology Thurgau (BITG) at the University of Konstanz, Kreuzlingen, Switzerland.*

*^8^ Theodor Kocher Institute, University of Bern, Bern, Switzerland*

*^9^ Department of Biology, University of Konstanz, Konstanz, Germany.*

*^10^ Immunology and General Pathology Laboratory, Department of Biotechnology and Life Sciences, University of Insubria, Varese, Italy*

*^11^ Unit of Molecular Pathology, Biochemistry and Immunology, Istituto di Ricovero e Cura a Carattere Scientifico (IRCCS) MultiMedica, Milan, Italy*

*^Ψ^Equal contribution.*

**Equal contribution.*

*^&^***Corresponding Author:** Santiago Fernandez Gonzalez, Institute for Research in Biomedicine, via Francesco Chiesa 5. CH-6500 Bellinzona. Switzerland. Tel: (+41) 91 820 03 60, Email: [santiago.gonzalez@irb.usi.ch](mailto:santiago.gonzalez@irb.usi.ch)

**This PDF includes:**

Figure S1 to S5

Tables S1

**Supplementary Figures**

***Suppl. Fig. 1. BNT162b2 vaccine contributes to the formation of an antibody response in mice. (A)*** *Experimental strategy. IVM, Intravital microscopy; FACS, fluorescence-activated cell sorting; ELISA, enzyme-linked immunosorbent assay.* ***(B)*** *A gating strategy was used to identify different populations of bnt162B2 vaccine^+^ leukocytes. B cells and pDCs were identified as CD45^+^/CD3^-^/B220^+^ cells, and they were distinguished by the expression of PDCA. Meanwhile, T cells were gated CD45^+^/B220^-^/CD3^+^ cells. Neutrophils were identified in the gate of CD45^+^/CD3^-^/B220^-^ as positive for GR1. Within the negative population, Medullary macrophages were gated as F4/80^+^/CD11b^+^. NK cells were identified as CD45^+^/CD3^-^/B220^-^/GR1^-^/F4/80^-^/NK1.1^+^. To conclude, in the CD45^+^/CD3^-^/B220^-^/GR1^-^/F4/80^-^/NK1.1^-^ it was possible to identify CD11b^-^ DCs as CD11c^+^/CD11b^-^, CD11b^+^ DCs as CD11c^+^/CD11b^+^, and medullary macrophages as CD11c^-^/CD11b^+^.* ***(C)*** *A gating strategy was used to identify different populations of spike^+^ leukocytes. The same gating strategy reported in* ***(B)*** *was used.* ***(D)*** *Systemic anti-SARS-CoV-2 RBD IgM titers were measured by ELISA on day 7 p.v.* ***(E)*** *Local anti-SARS-CoV-2 RBD IgM response measured by ELISPOT on day 10 p.v. Data are presented as mean ± SD. Mann-Whitney U test. (* p < 0.05, ** p < 0.01, *** p < 0.001 **** p<0.0001).*

***Suppl. Fig. 2. pLN activation following BNT162b2 vaccination. (A)*** *Flow cytometric analysis showing the count of pDCs in the LN at resting condition (Day 0) and one day (Day +1) or three days (Day +3) after aPDCA administration. (n=4 for Day +1 group, n=3 for all the other groups).* ***(B)*** *Area under the curve (AUC) for systemic anti-SARS-CoV-2 RBD IgM for WT and aPDCA-treated mice at day 7 p.v. (n=6 for each group). The graph represents two independent experiments. Data are presented as mean ± SD.* ***(C)*** *Histogram showing the neutralizing serum antibody titers (ND50) of WT compared to aPDCA-treated mice at day 21 p.v. (n=5 for each group). Data are presented as geometric mean ± 95% CI.* *Data are presented as mean ± SD. Student's t-test* ***(B)****. One-way ANOVA* ***(A)*** *followed by Bonferroni correction for multiple comparisons. (* p < 0.05, ** p < 0.01, *** p < 0.001 **** p<0.0001).*

***Suppl. Fig. 4. pDCs and CD8^+^ T cells gene expression in the pLN of vaccinated mice. (A)*** *Comparison of the significant ligand-receptor pairs between pDCs and CD4^+^ (left graph) or CD8^+^ (right graph) T cells in the dLN p.v. Dot color reflects communication probabilities, and dot size represents computed p-values. Space indicates that the communication probability is zero. p-values are calculated from one-sided permutation test.* ***(B)*** *Truncated violin plots showing gene expression of pDCs on the single-cell level at 0 h, 24 h, and 7 days p.v. Each dot represents a single cell.* ***(C)*** *Truncated* *violin plots showing gene expression of CD8^+^ T cells on the single-cell level at 0 h, 24 h, and 7 days p.v. Each dot represents a single cell.* ***(D)*** *Histogram showing the expression levels of CCL5 (left) and TNF-a (right) in the LN following administration of the BNT162b2 vaccine in WT animals, in a group treated with aPDCA and in IFNAR KO mice at 12 h p.v. (n=9 for WT and aPDCA group, n=10 for IFNAR KO group). The negative control group (PBS) is indicated with a dashed line. Data are presented as median ± SD. One-way ANOVA followed by Bonferroni correction for multiple comparisons (* p < 0.05, ** p < 0.01, *** p < 0.001 **** p<0.0001).*

***Suppl.Fig. 5. T cell phenotype in the pLN of vaccinated mice. (A)*** *plots showing single CD8^+^ T cells from specific sets of samples. The grey background refers to the total CD8^+^ T cells in the dataset.* ***(B)*** *The gating strategy was used to identify different subsets of CD8^+^ T cells, which were gated as single/viable/CD45^+^/CD3^+^/B220^-^/CD8^+^. Active proliferating and Active Central Memory (CM) CD8^+^ T cells were identified as Ki-67^+^/CD62L^+^, respectively negative or positive for CD44. Naïve and DN CD8^+^ T cells were identified as Ki-67^-^/CD69^-^, respectively positive or negative for CD62L. Finally, the last three subsets were commonly identified as Ki-67^-^/CD69^+^, and divided into Active Naïve CD8^+^ T cells CD62L^+^/CD44^-^, Active CM CD8^+^ T cells CD62L^+^/CD44^+^, and Active DN CD8^+^ T cells CD62L^-^/CD44^+^.* ***(C)*** *Histogram showing DN, Active CM, Proliferating CM CD8^+^ T cell subsets at 0 h, 24 h, or 7 days p.v. in the pLN. (n=3 for each group). Data are presented as mean ± SD. One-way ANOVA followed by Bonferroni correction for multiple comparisons (* p < 0.05, ** p < 0.01, *** p < 0.001 **** p<0.0001).*

**Supplementary Table**

**Table 1	Antibody list**

| **Antibody** | **Fluorophore** | **Company** | **Cat. Num** | **Clone** | **RRID** |
| --- | --- | --- | --- | --- | --- |
| F4/80 | FITC | Biolegend | 123120 | BM8 | AB_893479 |
| CD86 | PercCP | Biolegend | 105026 | GL-1 | AB_893417 |
| Gr1 | PE-CF594 | BD Bioscience | 562700 | 1A8 | AB_2737730 |
| CD11c | BV711 | Biolegend | 117349 | N418 | AB_2563905 |
| CD11b | BUV395 | BD Bioscience | 563553 | M1/70 | AB_2738276 |
| B220 | BUV496 | BD Bioscience | 612950 | RA3-6B2 | AB_2870227 |
| CD45 | BUV563 | BD Bioscience | 612924 | 30-F11 | AB_2870209 |
| NK1.1 | BUV615 | BD Bioscience | 751111 | PK136 | AB_2875140 |
| CD3 | PECY7 | Biolegend | 100220 | 17A2 | AB_1732057 |
| CD21/35 | Pacific Blue | Biolegend | 123414 | 7 E9 | AB_2085158 |
| CD11c | BV711 | Biolegend | 117349 | N418 | AB_2563905 |
| CD169 | AF647 | Biolegend | 142408 | 3D6.112 | AB_2563621 |
| CD16/32 | Purified | Biolegend | 101302 | 93 | AB_312801 |
| Zombie aqua fixable viability kit |  | Biolegend | 423101 |  |  |
| CD19 | BV605 | Biolegend | 115540 | 6D5 | AB_2563067 |
| IgG | PE-CF594 | BD Bioscience | 562538 | G18-145 | AB_2737640 |
| CD4 | BV786 | Biolegend | 100453 | GK1.5 | AB_2565843 |
| CD3 | PerCP-eFluor710 | ThermoFisher | 46-0032-80 | 17A2 | AB_1834428 |
| CD3 | BV711 | Biolegend | 100241 | 17A2 | AB_2563945 |
| PDCA | BV605 | Biolegend | 127025 | 927 | AB_2687121 |
| SARS-CoV-2 Spike S1 Subunit | BV750 | Biotechne | FAB105805S | 1035226 | AB_3645376 |
| SARS-CoV-2 Spike S1 Subunit | DyLight 594 | Biotechne | FAB105805M | 1035226 | AB_3110475 |
| PDCA | PE | Biolegend | 127009 | 927 | AB_1953284 |
| CD1d | BV421 | Biolegend | 123517 | 1B1 | AB_10679123 |
| CD43 | PE | BD Bioscience | 553271 | S7 | AB_394748 |
| IgM | PE-Cy7 | Biolegend | 406513 | RMM-1 | AB_10640069 |
| CD8 | BV650 | Biolegend | 100742 | 53-6.7 | AB_2563056 |
| CD44 | APC | Biolegend | 103017 | IM7 | AB_493680 |
| CD62L | PercP | Biolegend | 104430 | MEL-14 | AB_2187124 |
| CD69 | FITC | Biolegend | 104506 | H1.2F3 | AB_313109 |
| Ki-67 | PE | ThermoFisher | 12-5698-80 | SolA15 | AB_11149672 |
| MHC I | BV421 | Invitrogen | 48_9598_82 | AF6_88.5.5.3 |  |
| B220 | APC-H7 | Miltenyi | 130-110-849 | REA775 | AB_2658286 |
| CD103 | PercP Cy5.5 | Biolegend | 121416 | 2.00E+07 | AB_2128621 |
